# The value of the dual systems model of adolescent risk-taking

**DOI:** 10.3389/fnhum.2013.00223

**Published:** 2013-05-27

**Authors:** Nicole M. Strang, Jason M. Chein, Laurence Steinberg

**Affiliations:** Department of Psychology, Temple UniversityPhiladelphia, PA, USA

In recent years, a perspective on adolescent risk-taking derived from developmental neuroscience has become increasingly popular. This perspective, referred to as the “dual systems model” (Somerville et al., 2010; Steinberg, 2010) or sometimes the “maturational imbalance theory” (Casey et al., 2011), posits that increased risk-taking during adolescence is due to a combination of heightened reward sensitivity and immature impulse control, which are tied to the development of two brain systems that undergo significant change during this age period, but that develop along different timetables. One system, which has been called the “socioemotional” incentive processing system (Steinberg, 2010; Chein et al., 2011) or “ventral affective system” (Pfeifer and Allen, 2012), is localized mainly in the ventral striatum and ventromedial prefrontal cortex. The second system, referred to as the “cognitive control” system (Steinberg, 2010; Chein et al., 2011) or “prefrontal control system” (Pfeifer and Allen, 2012), is localized mainly in lateral prefrontal, parietal, and anterior cingulate cortices (Wager and Smith, 2003; Owen et al., 2005). Briefly, the dual systems (DS) perspective posits that risk-taking during mid-adolescence is the product of the heightened reactivity of the socioemotional system against a backdrop of still maturing cognitive control. With further maturation, the socioemotional system becomes less reactive and the cognitive control system becomes stronger and more efficient. Together, these changes lead to an increase in risk taking during adolescence followed by a decrease in risk taking as individuals move into adulthood.

A recent article (Pfeifer and Allen, 2012) critiques the DS model. The authors make three main points: (1) that the data do not support the DS model because there are too few studies assessing the relationship between development in each brain system and patterns of “real-world” behavior; (2) that activation of the socioemotional system is sometimes associated with adaptive functioning, that activation of the cognitive control system is sometimes associated with maladaptive functioning, and that these pieces of evidence are contrary to the DS model; and (3) that patterns of brain development are more complex than those described by the DS theory.

As proponents of the DS perspective on adolescent risk taking, we welcome the opportunity to respond to the Pfeifer and Allen critique. While, we agree that presentations of the model have sometimes oversimplified the evidence or overlooked inconsistencies in the literature, we believe that the framework continues to offer a useful model for understanding risky behavior in adolescence. In the absence of an alternative theoretical account, which Pfeifer and Allen do not offer, the DS model provides a useful heuristic for the formulation of testable hypotheses.

Pfeifer and Allen (2012) make several excellent points about the model's limitations and the challenges of mapping neuroimaging findings onto specific theoretical claims. However, in our view, there are three main shortcomings in their critique. First and foremost, the authors fail to acknowledge that there is considerable behavioral evidence consistent with the predictions of the DS model. Reward sensitivity follows an inverted U-shaped curve (Steinberg et al., 2009; Romer, 2010; Harden and Tucker-Drob, 2011). In a large behavioral study of 10- to 30-year-olds, participants' self-report indicated a peak in sensation-seeking during mid-adolescence (Steinberg et al., 2009), and on a gambling task, participants' behavior was most influenced by rewarding stimuli during this same age period (Cauffman et al., 2010). In contrast, impulse control increases gradually and linearly, and the peak in performance on tasks measuring capabilities like planning and response inhibition occurs subsequent to the peak in reward sensitivity. This linear trajectory has been demonstrated in self-reports of impulsive behavior in several large-scale studies (Steinberg et al., 2009; Harden and Tucker-Drob, 2011). Additionally there is compelling evidence from behavioral studies of cognitive control, which demonstrate that performance improves gradually over the course of adolescence and does not peak until late adolescence (Luna, 2009; Albert and Steinberg, 2011). Furthermore, both impulsivity (Verdejo-García et al., 2008) and reward/sensation-seeking (Galvan et al., 2007; Romer, 2010) are correlated with self-reported risk-taking.

Importantly, the brain systems presumed to mediate these constructs follow similar developmental trajectories. The remodeling of dopaminergic pathways connecting the ventral striatum to the PFC is most pronounced shortly after puberty, just before the rise in reward sensitivity (Spear, 2009; Luciana and Collins, 2012). In contrast, the prefrontal and parietal cortices, which are thought to support age-related improvements in cognitive control (Luna and Sweeney, 2001; Bunge et al., 2002; Astle and Scerif, 2009; Luna et al., 2010), are among the last brain regions to mature (Huttenlocher, 1990; Giedd et al., 1999; Bitan et al., 2006). The value of the DS theory is that it provides an integrated account for the observed changes in risk-taking, in the psychological constructs presumed to contribute to risk-taking, and in the structural and functional neural changes presumed to contribute to the psychological changes.

Pfeifer and Allen (2012) are correct that there are few studies that assess, within the same experimental sample, correlations among real-world behavior, associated psychological functioning, and the presumed structural and functional neural substrates of these phenomena—but this is surely not a shortcoming unique to this area of inquiry. Indeed, the neuroimaging literature as a whole includes very few studies with an adequate sample to support strong conclusions about brain-behavior correlations, and serious objections have been raised about the potential for spurious conclusions to arise when small sample sizes are used (Yarkoni, 2009; Vul and Pashler, 2012). Even when a substantial correlation actually exists between brain activation in some particular region and behavior in the population, an atypically large fMRI sample is needed for a fair chance of detecting the effect [e.g., With a correlation of *r* = 0.5, 60 participants would be needed to give an 80% chance of detecting the effect at a statistical threshold of *p* < 0.001 (Yarkoni, 2009)]. Given that developmental MRI studies almost universally have sample sizes of fewer than 40 participants per age group, evaluating the DS model based on how well-individual differences in brain activation predict real world behaviors is an unreasonable criterion. It is fair to say that the DS model has seldom been tested in predictions of real world behavior, but untested is not the same as inaccurate.

Pointing out that two different aspects of development (brain and behavior) follow similar trajectories is, admittedly, not evidence that one trajectory causes the other, but neither is it merely argument by analogy, as Pfeifer and Allen (2012) assert. It strikes us as more than coincidental that patterns of behavior in adolescence follow a developmental course matching that observed in brain systems presumed to undergird these behaviors. As it becomes possible and more common to collect data from larger samples, it will be possible to examine brain-behavior relations through individual differences analyses. We note, however, that real-world behaviors are undoubtedly subserved by interacting and distributed brain networks, not just individual brain regions, and there is not currently a consensus on an analytic approach for exploring correlations between activations in interactive networks and concomitant behavior. Moreover, real-world risk-taking is influenced by a wide array of contextual variables that cannot be controlled in the lab.

Second, the authors mischaracterize the DS model as built on the assumption that activation of the socioemotional system is always maladaptive and activation of the cognitive control system is always adaptive; to our knowledge no such assertions have ever been made by proponents of the DS view. Perhaps this assertion arises in the popular press, but we don't believe it is a characteristic of scientific writings that employ this framework. The DS model is agnostic with respect to whether the developmental changes in brain structure or function produce desirable or undesirable consequences. Even, if the neural changes of adolescence impel individuals to take more risks, not all risk taking is undesirable; indeed, one of the central propositions of the framework is that the heightened risk taking seen during adolescence is an evolutionarily adaptive phenomenon. The hyper-responsivity to reward that characterizes adolescents relative to children and adults in many studies of reward processing (Ernst et al., 2005; Galvan et al., 2006; Geier et al., 2010; van Leijenhorst et al., 2010a,b; Chein et al., 2011; Christakou et al., 2011; Padmanabhan et al., 2011; Smith et al., 2011; Somerville et al., 2011) is neither inherently maladaptive nor inherently adaptive; to quote an overused colloquialism, “it is what it is.” In some individuals this may lead to maladaptive sensation-seeking, or drug use, or unprotected sex. In others, it may lead to more intense attempts to win the admiration of others, earn money, or do well in school. Rewards come in many forms, some socially valued, and others undesirable. Similarly, we find no descriptions of the DS theory which assert that more cognitive control is always more adaptive. The improved ability to imagine the future seen among older adolescents permits individuals to make more informed decisions, but it also enables them to ruminate, sometimes to a degree that may lead to or exacerbate depression.

Third, Pfeifer and Allen (2012) point out that there are findings that run counter to predictions derived from the DS model. It is hard to think of any theory of human development for which there are no inconsistent findings; a complete absence of inconsistency in the literature is an unrealistic criterion against which to evaluate a theory's utility. Over time, findings that are inconsistent with other studies or with predictions derived from a particular theory inevitably arise. In evaluating these instances, the chief considerations should be (1) whether the inconsistencies in the theory can be explained through more nuanced analyses or by refining the theory; (2) whether the putative inconsistencies are incompatible with what the theory actually predicts, rather than with mischaracterizations of the theory; and (3) whether there are so many inconsistencies that the theory is no longer useful.

The question of whether reward sensitivity is heightened during adolescence illustrates the first of these points nicely. While, it had once appeared as if findings on this issue were all over the map (with some studies finding adolescent hypersensitivity to reward, some finding adolescent hyposensitivity, and others finding no age differences), an accumulation of evidence has made it clear that adolescents most often evince a greater response to rewarding stimuli as compared to adults (Ernst et al., 2005; Galvan et al., 2006; Geier et al., 2010; van Leijenhorst et al., 2010a,b; Chein et al., 2011; Christakou et al., 2011; Padmanabhan et al., 2011; Smith et al., 2011; Somerville et al., 2011). Further, recent analyses have shown that consideration of the stage of the reward processing under examination (Galvan, 2010; Geier et al., 2010) may provide an explanation for those few studies (Bjork et al., 2004, 2011) that find a different pattern of results.

The second issue regarding inconsistencies is illustrated by Pfeifer and Allen's (2012) assertion that the DS theory predicts that one should see greater activation of prefrontal regions with age. This is not a prediction that comes from the DS theory. The expected relationships between age and activation depend on the task demands. The DS model predicts that when a task and the method of analysis provide an index of the *tendency* to engage cognitive control, PFC activation should be weaker on average among adolescents relative to adults. This is generally what one finds, for instance, if one examines the subset of studies exploring age-dependent PFC activation during working memory task performance, as reviewed by Crone and Dahl (2012), for which the majority of studies demonstrate a pattern of increasing PFC activity with age. In contrast, when a task and the associated method of analysis provide an index of the *efficiency* with which control is achieved, then PFC activation would be expected to be greater on average for adolescents relative to adults. Of course, determining whether, a specific approach provides an index of tendency or efficiency is not a simple undertaking because even subtly varying factors (e.g., proportion of targets to lures, degree of proactive interference, stimulus, and feedback timing, shape of assumed hemodynamic model, etc.) are known to affect how a task functions. Engaging in careful task analysis, garnering support from converging methodologies (e.g., behavior, ERP, computational modeling), and running larger sample sizes to allow greater exploration of brain-behavior relationships, might be useful ways to overcome this challenge.

There is behavioral evidence that is consistent with the DS model's prediction that both more and less PFC activation (relative to adults) can reflect immaturity. Depending on the experimental context, behavioral improvements in cognitive control have been associated with both more and with less activation within cognitive control circuitry (Velanova et al., 2009; Luna et al., 2010). Evidently, neural “maturation” in this realm means something more complex than “more” or “less” activation. Although, more systematic study is needed, it is likely that improvements in cognitive control are supported primarily by increases in structural and functional connectivity across and between cortical and subcortical regions (Olesen et al., 2003; Liston et al., 2006; Vincent et al., 2008; Schmithorst and Yuan, 2010). We anticipate that more clarity regarding the relationship between age-related improvements in cognitive control and brain activation will emerge as methods for assessing structural and functional connectivity improve (Dosenbach et al., 2010).

Having new tools to investigate the relationship between brain and behavior may allow us to make important advances, but in the absence of a testable theory we can't expect that much will be learned. We believe the DS model still serves an important purpose, in that it yields specific and testable predictions. We further, contend that there are fewer caveats and contradictory findings than have been implied by Pfeifer and Allen (2012), and indeed that there are many other sources of affirmative evidence for the DS model. Because the DS model took its original form from behavioral science it is to be expected that the model is not yet fully specified with respect to patterns of brain function. There is a complex mapping between psychological constructs and their neural underpinnings, and we anticipate that further specification of the model will become possible as the field develops refined ways to integrate behavioral and neuroscientific sources of evidence. We welcome further attempts to confirm, or disconfirm, aspects of the DS model, as well as the introduction of alternative theories that might better account for the phenomenology of adolescent risk behavior.
